# Efficacy of first‐line treatment options beyond RET‐TKIs in advanced RET‐rearranged non‐small cell lung cancer: A multi‐center real‐world study

**DOI:** 10.1002/cam4.6960

**Published:** 2024-02-01

**Authors:** Yihui Ge, Juan Li, Wenjing Gong, Jian Wang, Xiaojuan Wei, Jing Liu, Shuyun Wang, Leirong Wang, Haifeng Sun, Qinglei Cheng, Yanxin Sun, Qi Dang, Yuping Sun, Aiqin Gao

**Affiliations:** ^1^ Phase I Clinical Research Center Shandong University Cancer Center Jinan China; ^2^ Phase I Clinical Research Center Shandong Cancer Hospital and Institute, Shandong First Medical University and Shandong Academy of Medical Sciences Jinan China; ^3^ Medical Department The Affiliated Yantai Yuhuangding Hospital of Qingdao University Yantai China; ^4^ Department of Medical Oncology Qilu Hospital of Shandong University Jinan China; ^5^ Department of Oncology The Affiliated Hospital of Qingdao University Qingdao China; ^6^ Department of Oncology Affiliated Hospital of Weifang Medical University Weifang P. R. China; ^7^ Weifang Medical University Weifang China; ^8^ Department of Thoracic Radiation Oncology Shandong University Cancer Center Jinan China

**Keywords:** combination schemes, efficacy, first‐line therapy, non‐small cell lung cancer, RET rearrangement

## Abstract

**Background:**

Although RET‐tyrosine kinase inhibitors (RET‐TKIs) are the preferred first‐line therapy for advanced RET‐arranged NSCLC, most patients cannot afford them. In this population, bevacizumab, immunotherapy, and chemotherapy are the most commonly used regimens. However, the optimal scheme beyond RET‐TKIs has not been defined in the first‐line setting.

**Methods:**

This retrospective study included 86 stage IV NSCLC patients harboring RET rearrangement from six cancer centers between May 2017 and October 2022. RET‐TKIs, chemotherapy, or one of the combination therapies (including immune checkpoint inhibitor (ICI) combined with chemotherapy (I + C), bevacizumab combined with chemotherapy (B + C), ICI and bevacizumab combined with chemotherapy (I + B + C)), were used as the first‐line therapeutics. The clinical outcomes and safety were evaluated.

**Results:**

Fourteen of the 86 patients received RET‐TKIs, 57 received combination therapies, and 15 received chemotherapy alone. Their medium PFS (mPFS) were 16.92 months (95% CI: 5.9–27.9 months), 8.7 months (95% CI: 6.5–11.0 months), and 5.55 months (95% CI: 2.4–8.7 months) respectively. Among all the combination schemes, B + C (*p* = 0.007) or I + B + C (*p* = 0.025) gave beneficial PFS compared with chemotherapy, while I + C treatment (*p* = 0.169) generated comparable PFS with chemotherapy. In addition, I + B + C treatment had a numerically longer mPFS (12.21 months) compared with B + C (8.74 months) or I + C (7.89 months) schemes. In terms of safety, I + B + C treatment led to the highest frequency of hematological toxicity (50%) and vomiting (75%), but no ≥G3 adverse effect was observed.

**Conclusions:**

I + B + C might be a preferred option beyond RET‐TKIs in the first‐line therapy of RET‐arranged NSCLC. Combination with Bevacizumab rather than with ICIs offered favorable survival compared with chemotherapy alone.

## Graphical abstract

Immunotherapy combined with bevacizumab and chemotherapy is a preferred option beyond RET‐TKIs in the first‐line therapy of RET‐arranged NSCLC, followed by the combination of bevacizumab and chemotherapy.

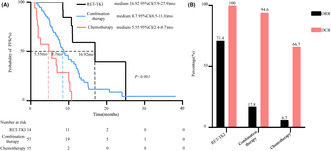

## 1 INTRODUCTION

Lung cancer is the leading cause of cancer‐related death worldwide, in which non‐small cell lung cancer (NSCLC) accounts for 80%–85%.^1^, ^2^ It is reported that more than half of the NSCLC patients are initially diagnosed at advanced stages, with a 5‐year survival rate of 15%–19%.^3^ Over the last three decades, the identification of oncogene mutations and the development of specific targeted therapies have greatly changed the treatment landscape of advanced NSCLC. For example, the application of *EGFR* tyrosine kinase inhibitors (EGFR‐TKIs) has extended overall survival (OS) by over 2 years, from an average of 10–12 months to 38.6 months.^4^, ^5^ Subsequently, a series of small‐molecule TKIs targeting specific oncogenic drivers, including *ALK*, *ROS1*, *KRAS*, *BRAF*, *MET*, and *NTRK*, were developed and approved as the preferred treatments for advanced NSCLC.^6^ These progresses have ushered in a “TKI first” era in NSCLC treatment.

The receptor tyrosine kinase rearranged during transfection (*RET*) gene is a proto‐oncogene encoding a transmembrane tyrosine kinase receptor that is crucial for normal embryonic development.^7^ *RET* alterations, including mutations, rearrangements, and amplifications, result in ligand‐independent constitutive activation of intracellular tyrosine kinases and downstream signaling pathway transmission, which subsequently promote tumor cell proliferation and invasion.^8^, ^9^ *RET* gene fusions occur in 1%–2% of NSCLC patients^10^, ^11^ and are usually mutually exclusive with *EGFR*, *KRAS*, *ALK*, or *ROS1* alterations. *RET* gene fusions are common in females, never‐smokers, and patients with lung adenocarcinomas. *RET* rearrangements are also associated with a high risk of brain metastases. It has been reported that 46% of NSCLC patients with *RET* rearrangements develop brain metastases during their lifetime.^12^ The most common fusion partners of *RET* rearrangements in NSCLC were *KIF5B* (71.2%) and *CCDC6* (16.85%).^13^

The treatment of RET‐rearranged NSCLC has been evolved from chemotherapy‐based combination therapy to multi‐kinase inhibitor (MKI) to selective RET‐tyrosine kinase inhibitors (RET‐TKI) in the past decades. Chemotherapy alone or combined with Bevacizumab are standard strategies before the appearance of targeted therapy.^14^ Platinum‐based chemotherapy generated an overall response rate (ORR) of approximately 50% and a medium progression‐free survival (PFS) of 6.4–9.2 months.^15^, ^16^ As the earliest targeted drugs approved by the United States Food and Drug Administration (FDA), MKIs, including cabozantinib, vandetanib, lenvantinib, and sorafenib, showed comparable clinical responses to chemotherapy but significantly increased toxicity.^17^, ^18^ In the last decade, immune checkpoint inhibitors (ICIs) have revolutionized NSCLC treatment in patients without oncogenic drivers. However, only a few retrospective studies with small sample sizes have reported the efficacy of ICIs in *RET*‐rearranged NSCLC.^13^, ^19^, ^20^ First‐line application of ICI‐based therapies displayed very different results among these studies, with an ORR of 20%–46%, and a PFS of 4.2–11.4 months. Recently, the IMpower150 treatment model (bevacizumab+ICIs+chemotherapy) was approved as the standard first‐line option for patients with NSCLC without oncogenic drivers or with sensitive *EGFR* mutations who are resistant to EGFR‐TKIs.^21^ However, the clinical benefits in patients with *RET* rearrangements are still undetermined. In the era of precision therapy, selective RET‐TKIs, including selpercatinib and pralsetinib, significantly improved the prognosis of advanced *RET*‐rearranged NSCLC as demonstrated in the LIBRETTO‐001 and ARROW studies.^22^, ^23^ The first‐line ORR and median PFS (mPFS) were 84% and 22 months, respectively, for selpercatinib, and 72% and 13 months, respectively, for pralsetinib. Based on these results, selpercatinib and pralsetinib are the preferred first‐line options among the above strategies.^14^ However, in real‐world practice, most patients do not choose RET‐TKIs as first‐line therapy because of their high cost or specific adverse events such as interstitial pneumonia. For these populations, bevacizumab, immunotherapy, chemotherapy, and their combinations are the standard regimens in the National Comprehensive Cancer Network (NCCN) or The Chinese Society of Clinical Oncology (CSCO) guidelines. However, an optimal scheme beyond RET‐TKIs has not yet been defined in the first‐line setting.

Here, we aimed to analyze the efficacy and safety of commonly used combination therapies as first‐line therapy for *RET*‐rearranged NSCLC. Our study provides real‐world evidence for clinical decision‐making in patients for whom RET‐TKIs cannot be selected.

## 2 MATERIALS AND METHODS

### 2.1 Inclusion criteria

Advanced NSCLC patients harboring *RET* rearrangements were retrospectively analyzed at six cancer institutions (Shandong Cancer Hospital and Institute, QiLu Hospital of Shandong University, Shandong Provincial Hospital, Yantai Yuhuangding Hospital, Affiliated Hospital of Qingdao University, and Jinan Central Hospital) in China between May 1, 2017, and October 31, 2022. The inclusion criteria were as follows: (1) confirmed NSCLC by cytology or histology; (2) stage IV disease according to the American Joint Committee on Cancer Staging Manual version 8; (3) confirmed *RET* rearrangement by fluorescence in situ hybridization, next‐generation sequencing, or polymerase chain reaction; and (4) patients receiving chemotherapy or chemotherapy‐based combination therapy (including ICI combined with chemotherapy [I + C], bevacizumab combined with chemotherapy [B + C], or ICI and bevacizumab combined with chemotherapy [I + B + C]) or RET‐TKIs as first‐line treatment. Patients with coexisting mutations (e.g., *EGFR* mutations) were excluded.

### 2.2 Data collection and tumor response assessment

Clinical, pathological, and molecular information of these patients, including age, sex, smoking history, histological types, metastasis sites, *RET* fusion partners, concomitant gene mutations, programmed death ligand‐1 (PD‐L1) expression, treatment regimens and duration, and adverse events, were recorded. Tumor response and progression were assessed using the Response Evaluation Criteria in Solid Tumors v1.1, stratified as complete response (CR), partial response (PR), stable disease (SD), or progressive disease (PD). The ORR was defined as the percentage of patients who achieved CR and PR. The sum of the CR, PR, and SD rates was defined as the disease control rate (DCR). PFS was calculated from the start of first‐line treatment to the date of disease progression or death from any cause. The final follow‐up was conducted on March 25, 2023. Adverse events (AEs) were assessed according to the National Cancer Institute Common Terminology Criteria for Adverse Events, version 5.0.

### 2.3 Statistical analysis

SPSS version 25.0 and GraphPad Prism 8 software were used for statistical analyses. Differences in ORR and DCR between the groups were calculated using the chi‐square test or Fisher's exact test. The Kaplan–Meier method with the log‐rank test was used to summarize and analyze survival rates. Subgroup, univariate, and multivariate survival analyses were performed using the Cox proportional hazards regression model. The Cox proportional hazards regression model was also applied to assess the hazard ratio (HR) and corresponding 95% confidence interval (95% CI). A *p* < 0.05 was considered statistically significant. A Bonferroni correction was used to compare multiple subgroups (more than two groups).

## 3 RESULTS

### 3.1 Clinical characteristics

Of the 86 patients with stage IV NSCLC and *RET* rearrangement, 65 (75.6%) were nonsmokers and 82 (95.2%) had adenocarcinoma. The clinical and pathological characteristics are summarized in Table 1. The most common sites of distant metastases were the bone (77.9%), lung (44.7%), and pleura (44.2%), followed by the liver and brain, with adrenal metastases being less common (Figure S1A). Brain metastases occurred in 17.4% of the patients at initial diagnosis and in 21.1% of the patients during treatment. Among the 30 known *RET* rearrangements, the most common fusion partner was *KIF5B* (63.3%, 19/30), followed by coiled‐coil domain‐containing 6 (*CCDC6*) (13.3%, 4/30) and nuclear receptor coactivator 4 (*NCOA4*) (6.7%, 2/30) (Figure S1B). The most common concurrent mutation was in *TP53* (14%; 12/86). A total of 38 individuals were tested for PD‐L1 expression, of which 18 (47.4%) had low PD‐L1 expression (PD‐L1 1%–49%), 10 (26.3%) had negative PD‐L1 expression (PD‐L1 < 1%), and 10 (26.3%) had high PD‐L1 levels (PD‐L1 ≥ 50%) (Figure S1C). Among the 86 included patients, 14 received RET‐TKI treatment (16.3%), 15 received chemotherapy (17.4%), and 57 (66.3%) received combination therapy (Figure S1D).

**TABLE 1:** Patient clinicopathological characteristics.

Characteristics	Patient (*n* = 86)
Age (years) *n* (%)	
<58	42 (48.8%)
≥58	44 (51.2%)
Gender *n* (%)	
Male	45 (52.3%)
Female	41 (47.7%)
Smoking history *n* (%)	
No	65 (75.6%)
Current or previous smoker	21 (24.4%)
Pathological type *n* (%)	
Adenocarcinoma	82 (95.2%)
Adenosquamous cell carcinoma	1 (1.2%)
Squamous cell carcinoma	2 (2.4%)
Large cell neuroendocrine	1 (1.2%)
With TP53 *n* (%)	12 (14.0%)

Abbreviation: *n*, number.

### 3.2 Efficacy of different first‐line options

**FIGURE 1:**
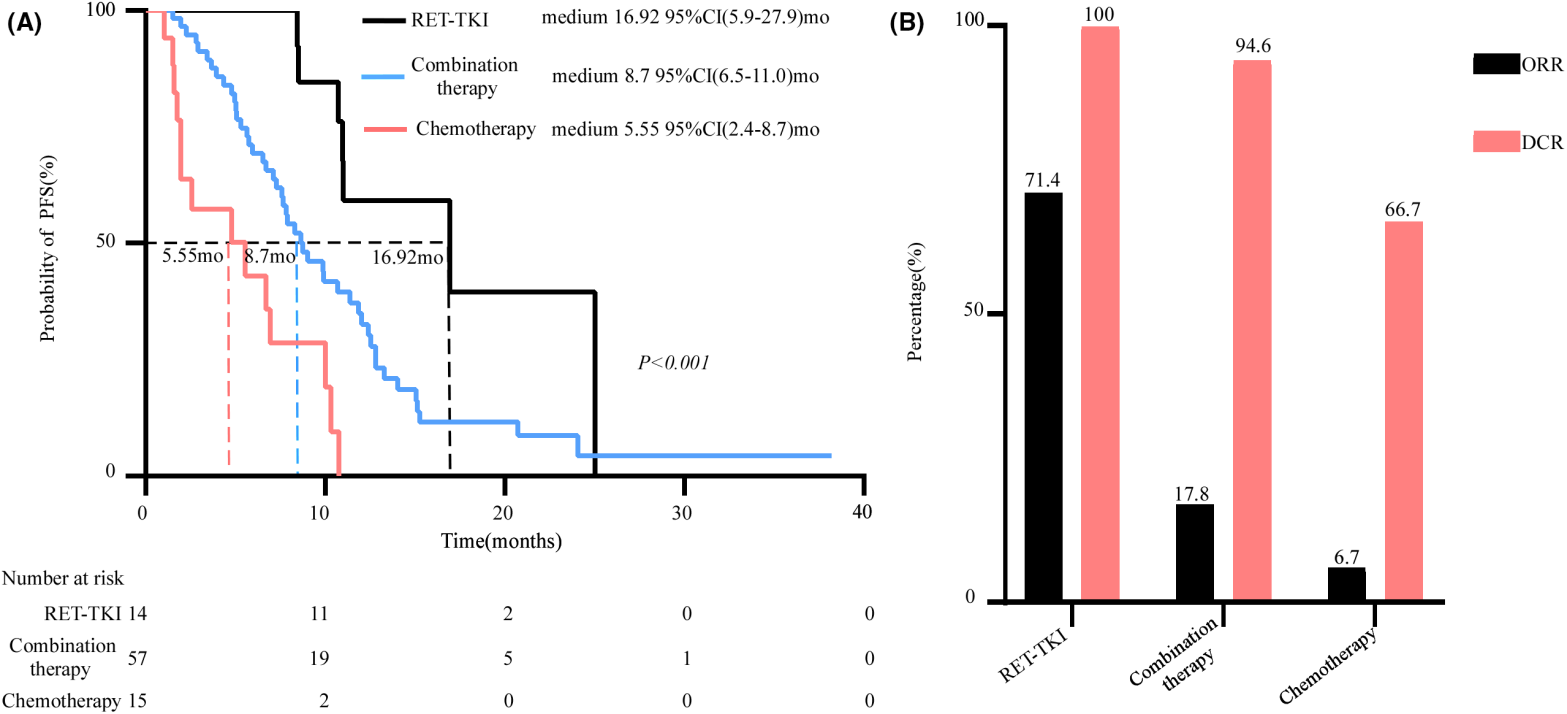
Efficacy of first‐line therapies. (A) The progression‐free survival (PFS) of RET‐rearranged NSCLC patients based on different first‐line therapies. The mPFS in patients receiving RET‐TKIs, chemotherapy‐based combination therapy, and chemotherapy alone were 16.92, 8.7, and 5.55 months, respectively. (B) The ORR and DCR of patients treated with different first‐line therapies. The ORRs were 71.4%, 17.8%, and 6.7% in patients receiving RET‐TKIs, combination therapy, and chemotherapy, respectively. RET‐TKIs, selective RET‐tyrosine kinase inhibitor; ORR, objective response rate; DCR, disease control rate.

First, we analyzed the efficacy of different first‐line options, including RET‐TKIs, chemotherapy, and chemotherapy‐based combination therapies. We found that RET‐TKIs and combination therapy generated longer PFS, with a mPFS of 16.92 months (95% CI: 5.9–27.9 months) and 8.7 months (95% CI: 6.5–11.0 months), respectively, compared with chemotherapy alone (mPFS = 5.55 months, 95% CI: 2.4–8.7 months) (Figure 1A). The ORR and DCR in patients receiving RET‐TKIs, combination therapy, and chemotherapy were 71.4% and 100%, 17.8% and 94.6%, and 6.7% and 66.7%, respectively (Figure 1B). These results confirmed that RET‐TKIs are the preferred option when available. But most patients did not choose RET‐TKIs as the first‐line therapy due to the high cost. Therefore, chemotherapy‐based combination therapy may be an alternative treatment strategy.

**FIGURE 2:**
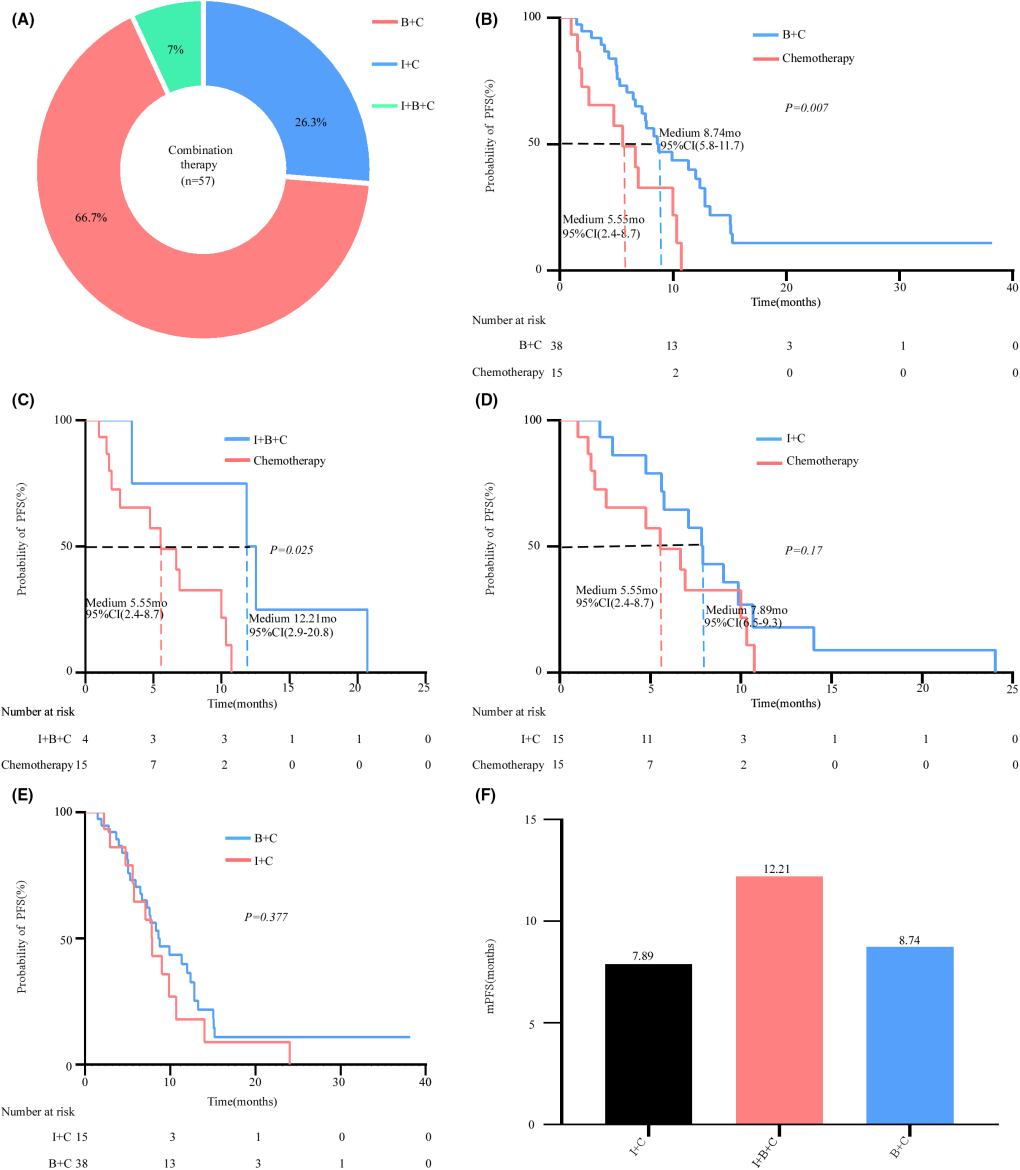
Efficacy of different combination therapies. (A) The frequency of patients receiving different combination therapies. 2/3 of patients received B + C and only 7% of patients received I + B + C treatment. (B) Patients treated with B + C had markedly longer progression‐free survival (PFS) compared with those with chemotherapy alone (*p* = 0.007). (C) Compared with chemotherapy, I + B + C treatment generated a longer PFS (*p* = 0.025). (D) I + C treatment generated numerical but not statistical improvement of PFS compared with chemotherapy alone (*p* = 0.17). (E) Treatment with I + C and B + C displayed comparable PFS (*p* = 0.377). (F) The mPFS (medium progression‐free survival) in I+B+C, B+C, and I+C group were 12.21, 8.74, and 7.89 months, respectively.

Because combination therapy showed beneficial PFS compared with chemotherapy, we further explored which combination scheme was better. Among the 57 patients in the combination therapy group, 15 (26.3%) received I + C, 38 (66.7%) received B + C, and 4 (7.0%) received I + B + C (Figure 2A). PFS was significantly longer in the B + C (*p* = 0.007) and I + B + C (*p* = 0.025) groups, but not in the I + C (*p* = 0.17) group, than in the chemotherapy group (Figure 2B–D). In addition, PFSs in the I + C and B + C groups were comparable (Figure 2E). The mPFS were 12.21, 8.74, and 7.89 months in I + B + C, B + C, and I + C groups, respectively (Figure 2F). These results indicated that the I + B + C treatment model may be a better choice for patients unsuitable for RET‐TKIs. The combination of chemotherapy and bevacizumab was superior to ICI treatment.

### 3.3 Safety

The treatment‐related AEs are shown in Table 2. The most common grade I/II AE in the RET‐TKI group was pneumonia (*n* = 3; 21.4%). Vomiting and hematologic toxicity were the most common grade I/II AEs in the chemotherapy and combination therapy groups. The I + B + C group had the highest incidence of vomiting, followed by the I + C and B + C groups. Hematologic toxicity normally appeared in the B + C group instead of the I + B + C and I + C groups, and the most common grade III/IV adverse events in chemotherapy and combination therapy were hematologic toxicity. Compared with the I + B + C and B + C groups, the I + C group had the highest incidence. Myocarditis occurred in two patients (13.3%) in the I + C group, resulting in discontinued ICI usage. AEs leading to treatment discontinuation did not occur in the I + B + C and B + C groups.

**TABLE 2:** Adverse events based on different treatment options.

	Grades 1–2					Grades 3–4				
	RET‐TKI (*n* = 14)	C (*n* = 15)	I + C (*n* = 15)	B + C (*n* = 38)	I + B + C (*n* = 4)	RET‐TKI (*n* = 14)	C (*n* = 15)	I + C (*n* = 15)	B + C (*n* = 38)	I + B + C (*n* = 4)
Hematological toxicity		2(13.3%)	1(6.7%)	6(15.8%)	1(25%)		1(6.7%)	4(26.7%)	3(7.9%)	1(25%)
Pneumonia	3(21.4%)									
Transaminases increased	2(14.3%)	1(6.7%)		5(13.2%)						
Fatigue			1(6.7%)							
Rash			1 (6.7%)	1 (2.6%)		1 (7.1%)		1 (6.7%)		
Myocarditis								2 (13.3%)		
Vomiting		4 (26.7%)	4 (26.7%)	6 (15.8%)	3 (75%)			1 (6.7%)		
Diarrhea	1 (7.1%)									
Dry mouth	1 (7.1%)									
Elevated blood creatine Phosphokinase	1 (7.1%)									
Hypoalbuminemia	2 (14.3%)									

Abbreviation: *n*, number.

### 3.4 The risk factors for PFS

Univariate and multivariate analyses were performed to identify the risk factors for PFS. In univariate analysis, smoking (*p* = 0.011), brain metastases (*p* = 0.009), liver metastases (*p* = 0.009), and adrenal metastases (*p* = 0.032) were associated with poor prognosis. In multivariate analysis, smoking (*p* = 0.031) and brain metastases (*p* = 0.018) were independent negative indicators of PFS (Table S1).

## 4 DISCUSSION

As a rare subtype of NSCLC, *RET* rearrangement predicts rapid disease progression and poor outcomes.^24^ Most patients are diagnosed at advanced stages of the disease, with young age and frequent central nervous system metastasis.^25^, ^26^ Timely and effective first‐line interventions are crucial for improving survival. The development of RET‐TKIs has changed the treatment paradigm in this subpolulation. It is reported selpercatinib and pralsetinib generated an ORR of 70%–85% and a intracranial response rate of 91% in the first‐line setting for patients at advanced stages.^23^, ^25^, ^26^, ^27^, ^28^ Although RET‐TKIs are the preferred first‐line options, most patients choose chemotherapy combined with bevacizumab and/or ICIs because of the high cost or specific AEs in real‐world data. In this population, no optimal scheme was defined. In this retrospective, multicenter study, we compared the efficacies of the most commonly used first‐line treatments, including RET‐TKIs, chemotherapy, and chemotherapy‐based combination regimens, including I + C, B + C, and I + B + C. Our results suggest that I + B + C might be a preferred option beyond RET‐TKIs as a first‐line therapy for *RET*‐rearranged NSCLC, followed by a combination of bevacizumab and chemotherapy. This study provides real‐world evidence for improved clinical decision‐making in first‐line settings.

Chemotherapy and chemotherapy‐based combination therapies, including I + C, B + C, or I + B + C, are commonly selected in clinical practice according to the recommendations of the NCCN or CSCO guidelines.^14^, ^29^ However, there is no evidence supporting the choice of these therapies. In the current study, treatment with I + B + C resulted in the highest ORR among these options and significantly improved PFS compared with chemotherapy, suggesting that I + B + C should be given priority when RET‐TKIs are unavailable. AEs in the I + B + C group were similar to those in the I + C and B + C groups. Vascular endothelial growth factor suppresses T‐cell‐mediated immune responses by inhibiting dendritic cell maturation, restricting T‐cell infiltration, and inducing suppressive cell components in the tumor microenvironment.^30^ In preclinical models, bevacizumab reverses the immunosuppressive tumor microenvironment and improves the efficacy of ICI.^31^ When used in patients with NSCLC, the combination of bevacizumab with ICIs and chemotherapy significantly prolonged patient survival compared with I + C or B + C in *EGFR*‐mutant but TKI‐resistant NSCLC.^32^ However, to the best of our knowledge, the efficacy of the combined I + B + C treatment in *RET*‐rearranged NSCLC has not yet been determined. Here, we provide the first insights into this field and demonstrate the superior benefits of combining bevacizumab and ICIs.

Immunotherapy has recently revolutionized the NSCLC treatment paradigm. However, its role in *RET*‐rearranged NSCLC remains unclear. In some retrospective studies, ICIs alone have poor efficacy, with an ORR of 6.3%–23% and a mPFS of 2.1–3.6 months.^13^, ^33^, ^34^, ^35^, ^36^ Chemotherapy combined with ICIs has been shown to achieve promising outcomes in upfront treatment of *RET*‐rearranged NSCLC.^37^ However, real‐world data from different groups are controversial. The ORRs ranged between 21% and 46%, and the mPFS ranged from 6.1 to 9.6 months.^13^, ^20^ In the current study, the combination of ICIs and chemotherapy resulted in numerical, but not statistical, improvements in PFS, suggesting that the scheme should be considered only in certain conditions. Mechanistically, *RET* rearrangement has been shown to lower tumor mutation burden and PD‐L1 expression,^38^ explaining their hyporesponsiveness to ICIs.

Surprisingly, patients treated with a combination of ICIs and chemotherapy had a high probability of myocarditis in our cohort. Immunotherapy‐related myocarditis occurred in less than 1% of patients in previous studies.^39^ However, because of the absence of consistent myocardial injury biomarkers and recognizable clinical symptoms, the actual frequency may be underestimated. Immune‐related AEs have rarely been reported in *RET*‐rearranged NSCLC. In the RET‐MAP study, only one patient (2.4%, 1/41) had pericardial effusion, and no immune‐related cardiac toxicity was discovered.^13^ Another study in which patients were treated with ICIs alone did not report cardiac toxicity.^36^ Although combined ICIs and chemotherapy increased overall and grade III–V cardiac toxicity in a meta‐analysis,^40^ the frequency of cardiotoxicity in *RET*‐rearranged NSCLC needs further exploration in a larger patient cohort. In addition, the occurrence of Grade I–II pneumonia in patients receiving Pralsetinib was 21.4% in our study, which is higher than 6% as reported in ARROW study.^22^ We also observed an earlier onset of pneumonia within 1–3 months after receiving RET‐TKI compared with 6 months in the previous study.^41^ We speculated strict patient screening in clinical trials might decrease the incidence of pneumonia. Also, it may be related to the small number of patient samples. These results suggest that RET‐TKI‐related pneumonia should be given early attention to avoid serious consequences.

Despite these meaningful findings, our study has some limitations. First, whether the extension of PFS in our study can be converted to a benefit in OS requires further follow‐up. Second, the superiority of the I + B + C therapy requires further confirmation with larger sample sizes and prospective studies. Third, due to the insufficient number of cases in the I + C group, we could not identify the subpopulation that could benefit from immunotherapy.

## 5 CONCLUSIONS

In conclusion, I + B + C might be a preferred option beyond RET‐TKIs as a first‐line therapy for patients with *RET*‐arranged NSCLC. Combination therapy with bevacizumab, rather than ICIs, resulted in favorable survival compared with chemotherapy alone.

## AUTHOR CONTRIBUTIONS

**Yihui Ge:** Data curation (equal); formal analysis (equal); resources (equal); software (equal); writing – original draft (equal). **Juan Li:** Data curation (equal); formal analysis (equal); resources (equal); software (equal); writing – original draft (equal). **Wenjing Gong:** Data curation (supporting); supervision (equal). **Jian Wang:** Data curation (equal). **Xiaojuan Wei:** Data curation (equal). **Jing Liu:** Data curation (equal). **Shuyun Wang:** Data curation (equal). **Leirong Wang:** Data curation (equal). **Haifeng Sun:** Data curation (equal). **Qinglei Cheng:** Data curation (equal). **Yanxin Sun:** Data curation (equal). **Qi Dang:** Validation (equal); visualization (equal). **Yuping Sun:** Conceptualization (equal); validation (equal); visualization (equal). **Aiqin Gao:** Conceptualization (equal); data curation (equal); formal analysis (equal); software (equal); writing – review and editing (equal).

## FUNDING INFORMATION

This work was supported by grants from the National Natural Science Foundation of China (82103340), Natural Science Foundation of Shandong Province (ZR2021MH268), China Postdoctoral Science Foundation (2021M700054), and the Jinan Science and Technology Innovation Program of Clinical Medicine (202134041 and 202225015).

## CONFLICT OF INTEREST STATEMENT

All authors have completed the ICMJE uniform disclosure form. The authors have no conflicts of interest to declare.

## ETHICS STATEMENT

The author is responsible for all aspects of the work to ensure that issues are appropriately investigated and resolved. This study was approved by the Shandong Cancer Hospital and Institute review board (SDTHEC2023007007). The requirement for informed consent was waived because our study was retrospective without information disclosure and we did not hurt patient health. Our study did not involve any animal experiments.

## Supporting information


Data S1.

## Data Availability

Due to the nature of this research, participants of this study did not agree for their data to be shared publicly, so supporting data are not available.

## References

[1] Sung H , Ferlay J , Siegel RL , et al. Global cancer statistics 2020: GLOBOCAN estimates of incidence and mortality worldwide for 36 cancers in 185 countries. CA Cancer J Clin. 2021;71(3):209‐249. 33538338 10.3322/caac.21660

[2] Duma N , Santana‐Davila R , Molina JR . Non‐small cell lung cancer: epidemiology, screening, diagnosis, and treatment. Mayo Clin Proc. 2019;94(8):1623‐1640. 31378236 10.1016/j.mayocp.2019.01.013

[3] Herbst RS , Morgensztern D , Boshoff C . The biology and management of non‐small cell lung cancer. Nature. 2018;553(7689):446‐454. 29364287 10.1038/nature25183

[4] Spiro SG , Silvestri GA . One hundred years of lung cancer. Am J Respir Crit Care Med. 2005;172(5):523‐529. 15961694 10.1164/rccm.200504-531OE

[5] Ramalingam SS , Vansteenkiste J , Planchard D , et al. Overall survival with Osimertinib in untreated, EGFR‐mutated advanced NSCLC. N Engl J Med. 2020;382(1):41‐50. 31751012 10.1056/NEJMoa1913662

[6] Chen R , Manochakian R , James L , et al. Emerging therapeutic agents for advanced non‐small cell lung cancer. J Hematol Oncol. 2020;13(1):58. 32448366 10.1186/s13045-020-00881-7 PMC7245927

[7] Stencel K , Chmielewska I , Milanowski J , Ramlau R . Non‐small‐cell lung cancer: new rare targets‐new targeted therapies‐state of the art and future directions. Cancers (Basel). 2021;13(8):1829. DOI: 10.3390/cancers13081829. 33921237 PMC8070470

[8] Kato S , Subbiah V , Marchlik E , Elkin SK , Carter JL , Kurzrock R . RET aberrations in diverse cancers: next‐generation sequencing of 4,871 patients. Clin Cancer Res. 2017;23(8):1988‐1997. 27683183 10.1158/1078-0432.CCR-16-1679

[9] Subbiah V , Cote GJ . Advances in targeting RET‐dependent cancers. Cancer Discov. 2020;10(4):498‐505. 32094155 10.1158/2159-8290.CD-19-1116 PMC7125013

[10] Griesinger F , Eberhardt W , Nusch A , et al. Biomarker testing in non‐small cell lung cancer in routine care: analysis of the first 3717 patients in the German prospective, observational, nation‐wide CRISP registry (AIO‐TRK‐0315). Lung Cancer. 2021;152:174‐184. 33358484 10.1016/j.lungcan.2020.10.012

[11] Tan AC , Tan DSW . Targeted therapies for lung cancer patients with oncogenic driver molecular alterations. J Clin Oncol. 2022;40(6):611‐625. 34985916 10.1200/JCO.21.01626

[12] Drilon A , Lin JJ , Filleron T , et al. Frequency of brain metastases and multikinase inhibitor outcomes in patients with RET‐rearranged lung cancers. J Thorac Oncol. 2018;13(10):1595‐1601. 30017832 10.1016/j.jtho.2018.07.004 PMC6434708

[13] Aldea M , Marinello A , Duruisseaux M , et al. RET‐MAP: an international multicenter study on clinicobiologic features and treatment response in patients with lung cancer harboring a RET fusion. J Thorac Oncol. 2023;18(5):576‐586. 36646211 10.1016/j.jtho.2022.12.018

[14] Non‐Small Cell Lung Cancer . Version 3.2022, NCCN Clinical Practice Guidelines in Oncology. 2022. 10.6004/jnccn.2022.0025 35545176

[15] Gautschi O , Milia J , Filleron T , et al. Targeting RET in patients with RET‐rearranged lung cancers: results from the global, multicenter RET registry. Journal of Clinical Oncology. 2017;35(13):1403‐1410. 28447912 10.1200/JCO.2016.70.9352 PMC5559893

[16] Shen T , Pu X , Wang L , et al. Association between RET fusions and efficacy of pemetrexed‐based chemotherapy for patients with advanced NSCLC in China: a multicenter retrospective study. Clin Lung Cancer. 2020;21(5):e349‐e354. 32143967 10.1016/j.cllc.2020.02.006

[17] Drilon A , Hu ZI , Lai GGY , Tan DSW . Targeting RET‐driven cancers: lessons from evolving preclinical and clinical landscapes. Nat Rev Clin Oncol. 2018;15(3):151‐167. 29134959 10.1038/nrclinonc.2017.175 PMC7938338

[18] Sankar K , Gadgeel SM , Qin A . Molecular therapeutic targets in non‐small cell lung cancer. Expert Rev Anticancer Ther. 2020;20(8):647‐661. 32580596 10.1080/14737140.2020.1787156

[19] Bhandari NR , Hess LM , Han Y , Zhu YE , Sireci AN . Efficacy of immune checkpoint inhibitor therapy in patients with RET fusion‐positive non‐small‐cell lung cancer. Immunotherapy. 2021;13(11):893‐904. 34139897 10.2217/imt-2021-0035

[20] Meng Y , Yang Y , Fang Y , et al. The treatment status of patients in NSCLC with RET fusion under the prelude of selective RET‐TKI application in China: a multicenter retrospective research. Front Oncol. 2022;12:864367. 35692799 10.3389/fonc.2022.864367 PMC9176213

[21] Socinski MA , Jotte RM , Cappuzzo F , et al. Atezolizumab for first‐line treatment of metastatic nonsquamous NSCLC. N Engl J Med. 2018;378(24):2288‐2301. 29863955 10.1056/NEJMoa1716948

[22] Griesinger F , Curigliano G , Thomas M , et al. Safety and efficacy of pralsetinib in RET fusion‐positive non‐small‐cell lung cancer including as first‐line therapy: update from the ARROW trial. Ann Oncol. 2022;33(11):1168‐1178. 35973665 10.1016/j.annonc.2022.08.002

[23] Drilon A , Subbiah V , Gautschi O , et al. Selpercatinib in patients with RET fusion‐positive non‐small‐cell lung cancer: updated safety and efficacy from the registrational LIBRETTO‐001 phase I/II trial. J Clin Oncol. 2023;41(2):385‐394. 36122315 10.1200/JCO.22.00393 PMC9839260

[24] Lu C , Dong XR , Zhao J , et al. Association of genetic and immuno‐characteristics with clinical outcomes in patients with RET‐rearranged non‐small cell lung cancer: a retrospective multicenter study. J Hematol Oncol. 2020;13(1):37. 32295619 10.1186/s13045-020-00866-6 PMC7160902

[25] Ortega MA , Pekarek L , Navarro F , et al. Updated views in targeted therapy in the patient with non‐small cell lung cancer. J Pers Med. 2023;13(2):167. 36836402 10.3390/jpm13020167 PMC9959016

[26] Ortega MA , Navarro F , Pekarek L , et al. Exploring histopathological and serum biomarkers in lung adenocarcinoma: clinical applications and translational opportunities (review). Int J Oncol. 2022;61(6):154. 36263628 10.3892/ijo.2022.5444 PMC9635864

[27] Gainor JF , Curigliano G , Kim DW , et al. Pralsetinib for RET fusion‐positive non‐small‐cell lung cancer (ARROW): a multi‐cohort, open‐label, phase 1/2 study. Lancet Oncol. 2021;22(7):959‐969. 34118197 10.1016/S1470-2045(21)00247-3

[28] Subbiah V , Gainor JF , Oxnard GR , et al. Intracranial efficacy of Selpercatinib in RET fusion‐positive non‐small cell lung cancers on the LIBRETTO‐001 trial. Clin Cancer Res. 2021;27(15):4160‐4167. 34088726 10.1158/1078-0432.CCR-21-0800 PMC8447251

[29] The Chinese Society of Clinical Oncology (CSCO). Non‐Small Cell Lung Cancer; 2023.

[30] Chen DS , Hurwitz H . Combinations of bevacizumab with cancer immunotherapy. Cancer J (Sudbury, Mass). 2018;24(4):193‐204. 10.1097/PPO.0000000000000327 30119083

[31] Jain RK . Antiangiogenesis strategies revisited: from starving tumors to alleviating hypoxia. Cancer Cell. 2014;26(5):605‐622. 25517747 10.1016/j.ccell.2014.10.006 PMC4269830

[32] Reck M , Mok TSK , Nishio M , et al. Atezolizumab plus bevacizumab and chemotherapy in non‐small‐cell lung cancer (IMpower150): key subgroup analyses of patients with EGFR mutations or baseline liver metastases in a randomised, open‐label phase 3 trial. Lancet Respir Med. 2019;7(5):387‐401. 30922878 10.1016/S2213-2600(19)30084-0

[33] Hegde A , Andreev‐Drakhlin AY , Roszik J , et al. Responsiveness to immune checkpoint inhibitors versus other systemic therapies in RET‐aberrant malignancies. ESMO Open. 2020;5(5):e000799. 33097651 10.1136/esmoopen-2020-000799 PMC7590373

[34] Offin M , Guo R , Wu SL , et al. Immunophenotype and response to immunotherapy of RET‐rearranged lung cancers. JCO Precis Oncol. 2019;3:3‐8. 10.1200/PO.18.00386 PMC6561651 31192313

[35] Mazieres J , Drilon A , Lusque A , et al. Immune checkpoint inhibitors for patients with advanced lung cancer and oncogenic driver alterations: results from the IMMUNOTARGET registry. Ann Oncol. 2019;30(8):1321‐1328. 31125062 10.1093/annonc/mdz167 PMC7389252

[36] Lee J , Ku BM , Shim JH , et al. Characteristics and outcomes of RET‐rearranged Korean non‐small cell lung cancer patients in real‐world practice. Jpn J Clin Oncol. 2020;50(5):594‐601. 32083304 10.1093/jjco/hyaa019

[37] Mathew M , Enzler T , Shu CA , Rizvi NA . Combining chemotherapy with PD‐1 blockade in NSCLC. Pharmacol Ther. 2018;186:130‐137. 29352857 10.1016/j.pharmthera.2018.01.003

[38] Ricciuti B , Wang X , Alessi JV , et al. Association of high tumor mutation burden in non‐small cell lung cancers with increased immune infiltration and improved clinical outcomes of PD‐L1 blockade across PD‐L1 expression levels. JAMA Oncol. 2022;8(8):1160‐1168. 35708671 10.1001/jamaoncol.2022.1981 PMC9204620

[39] Johnson DB , Balko JM , Compton ML , et al. Fulminant myocarditis with combination immune checkpoint blockade. N Engl J Med. 2016;375(18):1749‐1755. 27806233 10.1056/NEJMoa1609214 PMC5247797

[40] Liu S , Gao W , Ning Y , et al. Cardiovascular toxicity with PD‐1/PD‐L1 inhibitors in cancer patients: a systematic review and meta‐analysis. Front Immunol. 2022;13:908173. 35880172 10.3389/fimmu.2022.908173 PMC9307961

[41] Gao M , Zhang X , Yan H , et al. Pralsetinib‐associated pneumonia in RET fusion‐positive non‐small cell lung cancer. Support Care Cancer. 2023;31(12):671. 37924363 10.1007/s00520-023-08125-3 PMC10625509

