# Supplementary material for: Efficacy of first‐line treatment options beyond RET‐TKIs in advanced RET‐rearranged non‐small cell lung cancer: A multi‐center real‐world study
Source: Cancer Med. 2024 Feb 1;13(2):e6960. doi: 10.1002/cam4.6960 (PMC10832335; doi:10.1002/cam4.6960)
Supplement: Supplementary file 1 — Data S1. [file CAM4-13-e6960-s001.docx]

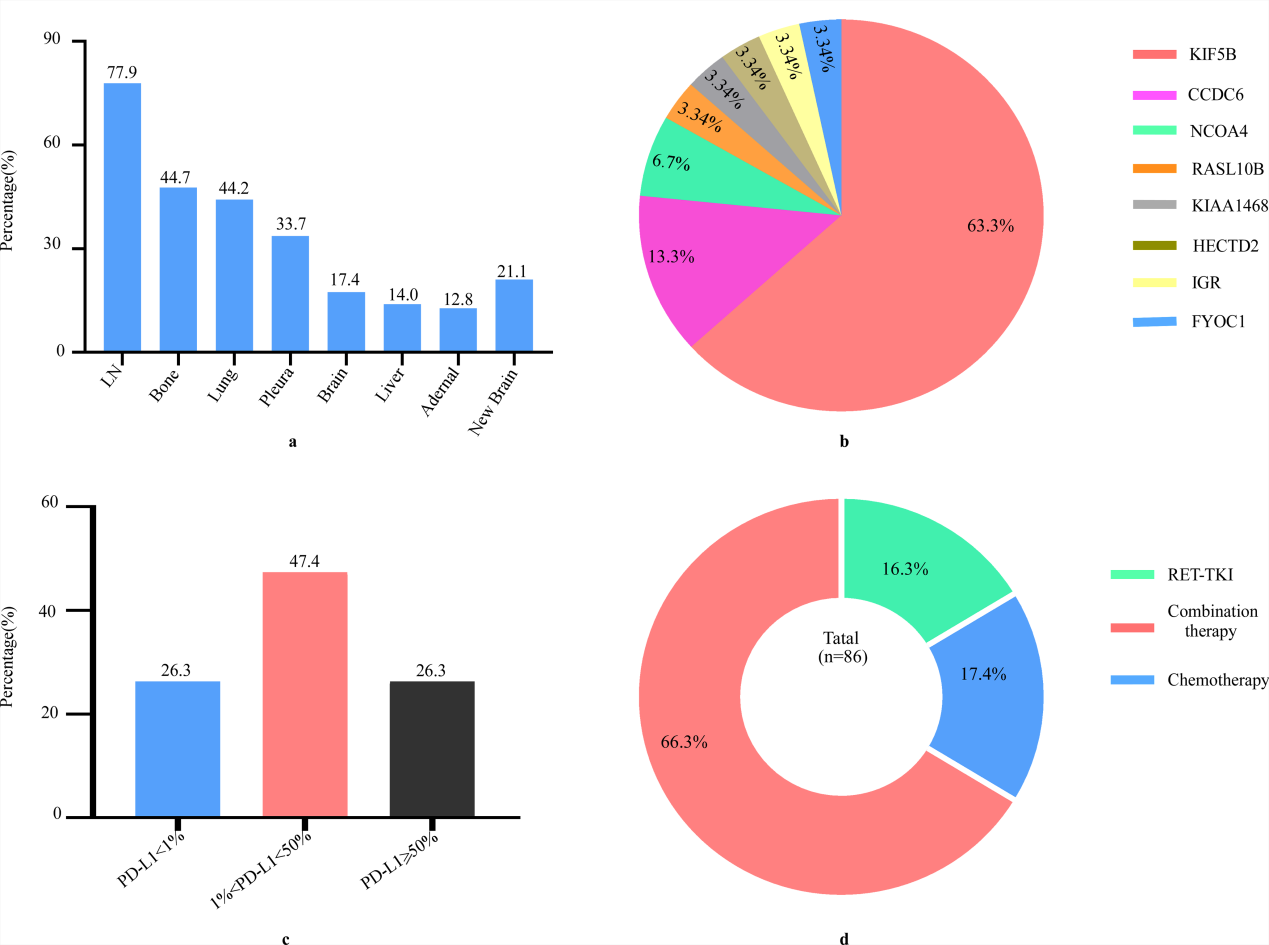


**Supplement Figure. Clinical and molecular characteristics of enrolled patients.**

1. Metastatic pattern of RET-rearranged stage IV NSCLC in 86 patients. The most common metastatic sites were bone, lung, and pleura, followed by liver and brain. **(B)** Frequency of RET rearrangement according to the fusion partners. The most frequent fusion partner was KIF5B. **(C)** The distribution of PD-L1 expression levels. Most RET-rearranged patients have low PD-L1 expression (47.4%). **(D)** The frequency of first-line treatment schemes in enrolled patients. LN: lymph nodes, New brain metastases: new developed brain metastases during treatment.

**Supplement Table. The risk factors for PFS by univariate and multivariate analysis.**

| Factors | Univariate analysis | | Multivariate analysis | |
| --- | --- | --- | --- | --- |
|  | HR(95%CI) | P-value | HR(95%CI) | P-value |
| Age |  |  |  |  |
| <58 | 1 | | 1 | |
| ≥58 | 1.443(0.881-2.365) | 0.145 | 1.906(1.033-3.516) | 0.039 |
| Gender |  |  |  |  |
| Male | 1 | |  |  |
| Female | 0.807(0.493-1.321) | 0.394 |  |  |
| Smoking history |  |  |  |  |
| NO | 1 | | 1 | |
| YES | **2.077(1.182-3.648)** | **0.011** | **2.013(1.068-3.796)** | **0.031** |
| Brain Metastases |  |  |  |  |
| NO | 1 | | 1 | |
| YES | **2.33(1.232-4.409)** | **0.009** | **2.335(1.155-4.721)** | **0.018** |
| Liver Metastases |  |  |  |  |
| NO | 1 | | 1 | |
| YES | **2.335(1.232-4.427)** | **0.009** | 2.102(1.002-4.411) | 0.05 |
| Bone Metastases |  |  |  |  |
| NO | 1 | | 1 | |
| YES | 1.439(0.871-2.377) | 0.155 | 1.067(0.608-1.874) | 0.821 |
| Pleura Metastases |  |  |  |  |
| NO | 1 | | 1 | |
| YES | 0.601(0.349-1.037) | 0.067 | 0.53(0.266-1.056) | 0.071 |
| Pulmonary Metastases |  |  |  |  |
| NO | 1 | | 1 | |
| YES | 0.666(0.402-1.103) | 0.115 | 1.58(0.873-2.86) | 0.131 |
| Lymph node Metastases |  |  |  |  |
| NO | 1 | | 1 | |
| YES | 1.627(0.846-3.126) | 0.144 | 2.342(1.127-4.87) | 0.023 |
| Adrenal Metastases |  |  |  |  |
| NO | 1 | | 1 | |
| YES | **2.123(1.065-4.231)** | **0.032** | 2.071(0.946-4.534) | 0.069 |
| KIF5B |  |  |  |  |
| NO | 1 | |  |  |
| YES | 1.312(0.728-2.363) | 0.366 |  |  |
| Treatment |  |  |  |  |
| Chemotherapy | 1 | | 1 | |
| Combination therapy | 0.374(0.192-0.731) | 0.004 | **0.236(0.11-0.508)** | **<0.001** |
| RET-TKI | 0.137(0.052-0.361) | <0.001 | **0.070(0.23-0.218)** | **<0.001** |

HR: Hazard ratio; Combination therapy: immune checkpoint inhibitor (ICI) combined with chemotherapy (I+C), Bevacizumab combined with chemotherapy (B+C), ICI and Bevacizumab combined with chemotherapy (I+B+C); RET-TKI: selective RET-tyrosine kinase inhibitor
